# User Experience With Dynamic Difficulty Adjustment Methods for an Affective Exergame: Comparative Laboratory-Based Study

**DOI:** 10.2196/25771

**Published:** 2021-05-31

**Authors:** Ali Darzi, Sean M McCrea, Domen Novak

**Affiliations:** 1 Department of Electrical and Computer Engineering University of Wyoming Laramie, WY United States; 2 Department of Psychology University of Wyoming Laramie, WY United States

**Keywords:** affective computing, dynamic difficulty adaptation, exergames, physiological measurements, task performance, personality characteristics, psychophysiology

## Abstract

**Background:**

In affective exergames, game difficulty is dynamically adjusted to match the user’s physical and psychological state. Such an adjustment is commonly made based on a combination of performance measures (eg, in-game scores) and physiological measurements, which provide insight into the player’s psychological state. However, although many prototypes of affective games have been presented and many studies have shown that physiological measurements allow more accurate classification of the player’s psychological state than performance measures, few studies have examined whether dynamic difficulty adjustment (DDA) based on physiological measurements (which requires additional sensors) results in a better user experience than performance-based DDA or manual difficulty adjustment.

**Objective:**

This study aims to compare five DDA methods in an affective exergame: manual (player-controlled), random, performance-based, personality-performance–based, and physiology-personality-performance–based (all-data).

**Methods:**

A total of 50 participants (N=50) were divided into five groups, corresponding to the five DDA methods. They played an exergame version of Pong for 18 minutes, starting at a medium difficulty; every 2 minutes, two game difficulty parameters (ball speed and paddle size) were adjusted using the participant’s assigned DDA method. The DDA rules for the performance-based, personality-performance–based, and all-data groups were developed based on data from a previous open-loop study. Seven physiological responses were recorded throughout the sessions, and participants self-reported their preferred changes to difficulty every 2 minutes. After playing the game, participants reported their in-game experience using two questionnaires: the Intrinsic Motivation Inventory and the Flow Experience Measure.

**Results:**

Although the all-data method resulted in the most accurate changes to ball speed and paddle size (defined as the percentage match between DDA choice and participants’ preference), no significant differences between DDA methods were found on the Intrinsic Motivation Inventory and Flow Experience Measure. When the data from all four automated DDA methods were pooled together, the accuracy of changes in ball speed was significantly correlated with players’ enjoyment (*r*=0.38) and pressure (*r*=0.43).

**Conclusions:**

Although our study is limited by the use of a between-subjects design and may not generalize to other exergame designs, the results do not currently support the inclusion of physiological measurements in affective exergames, as they did not result in an improved user experience. As the accuracy of difficulty changes is correlated with user experience, the results support the development of more effective DDA methods. However, they show that the inclusion of physiological measurements does not guarantee a better user experience even if it yields promising results in offline cross-validation.

## Introduction

### Affective Exergames

Exercise games (commonly shortened to exergames) are used to promote enjoyable, intensive exercise in applications such as weight loss and maintenance [1,2], healthy aging [3], and motor rehabilitation [4,5]. In such exergames, tailoring the game difficulty to the player’s abilities and preferences can often improve the user experience, enabling more enjoyable, frequent, and intensive exercise. Such difficulty adjustment can always be performed manually by a player via an interface. However, although manual adjustment is generally accurate, it can cause interruptions in game flow, as users must stop playing the game to adjust difficulty [6]. This has led to the development of dynamic difficulty adjustment (DDA) methods, where the game assesses the player’s current state and automatically adjusts the difficulty to bring the player into a more desirable state.

The simplest and most popular DDA methods are based only on performance measures (eg, in-game score), which provide an easily measurable and interpretable indicator of perceived game difficulty. However, although many studies have shown the positive effects of performance-based DDA on user experience [7], performance alone does not necessarily provide insight into the player’s psychological state, for example, a frustrated player can get a high score in a game without enjoying it. This has led to the development of affective games, an emerging type of videogame that adapts difficulty based on a combination of the player's performance and their psychological (cognitive and affective) state. This psychological state can be defined as, for example, the level of anxiety [8,9] or via the two-dimensional valence-arousal model [9,10] and is commonly inferred from measurements such as an electrocardiogram (ECG), galvanic skin response, and electroencephalography (EEG) using automated classification algorithms. Once the psychological state has been classified, DDA can be performed using rules such as “if performance is high and anxiety is low, increase difficulty.” Thus, incorporating psychological information allows affective games to potentially achieve more effective and personalized DDA than performance-based methods [11].

Affective exergames are most prominent in motor rehabilitation, where physiological measurements have been used to estimate psychological states and adjust the difficulty of exercises for both the upper [5,12-16] and lower limbs [17,18]; they have also been used for general exercise enhancement in several studies [19,20]. However, as affective exergames are more complex than simple performance-based DDA, the question arises: does the additional cost and complexity result in a better user experience?

### Do Affective Exergames Improve User Experience?

As mentioned, in affective exergames, psychological states are extracted from measurements such as ECG, EEG, and galvanic skin response using signal processing and machine learning techniques. The ground truth for such machine learning is generally the user’s self-reported state or opinion, for example, how anxious they are or how they would like difficulty to be adjusted [8,11,21]. The *accuracy* of DDA is then estimated as the percentage of times that the affective exergame makes the same DDA decision (based on the extracted psychological state) as the user would. This accuracy is never perfect; however, to justify the use of affective games, affective DDA should reach a higher accuracy than performance-based DDA and result in a better user experience than performance-based DDA.

Many studies in both affective exergaming and other fields of affective computing have shown that the addition of physiological measurements allows for a more accurate classification of the player’s psychological state compared with using only performance measurements [12,17,22-24]. Furthermore, some studies have shown that affective exergames that perform DDA based on physiological measurements achieve a positive user experience [5,13-15]. However, there is little evidence that the user experience achieved with affective exergames is better than that achieved with a performance-based exergame in the same context. To the best of our knowledge, only one study has examined this difference and has found that an affective exergame was more engaging than an equivalent performance-based exergame [14]. Even outside affective exergaming, the evidence in favor of affective DDA is limited, with a few studies in entertainment games showing better user experience with affective DDA than performance-based DDA [8] and a few studies showing a better user experience with affective DDA than manual (user-guided) DDA [25,26], although the user’s decisions are frequently used as the ground truth for training affective DDA.

This lack of evidence showing that affective technologies result in a better user experience than simpler technologies has been acknowledged as a significant issue in affective computing [27,28], as calculating the accuracy of psychological state classification or demonstrating a positive user experience with affective DDA may not be enough to show the superiority of affective technologies. For example, our own *Wizard of Oz* studies in affective games have shown that, although user psychological state classification accuracy is correlated with user experience, the relationship between the two is complex and nonlinear [29,30]. Furthermore, studies from other fields of human-machine interaction indicate that offline classification accuracy does not always strongly correlate with user experience or even with real-time (dynamic) classification accuracy [31,32]. Thus, lessons learned from offline classification studies cannot be directly transferred to physiology-based DDA. The state of the art in affective exergaming can be summarized as follows: there is evidence that affective exergames result in a positive user experience and that physiology-based psychological state classification is more accurate than performance-based psychological state classification. However, there is very limited evidence that physiology-based DDA (used in affective exergames) results in a better user experience than performance-based DDA (used in simpler exergames). Additional evidence in this regard is needed to justify the broader adoption of affective exergames.

### Goal of the Study

This study aims to evaluate the user experience in an exergame where DDA is performed using one of five methods: manual, random, performance-based (PE), personality-performance–based (PEPE), and physiology-personality-performance–based (all-data). The last three DDA methods were based on classifiers developed for offline psychological state classification in our previous study [33]. In this study, these classifiers were connected to *if-then* difficulty adjustment rules and used as a basis for DDA. The research questions were as follows:

- Research question 1: do the PEPE and all-data methods result in a better user experience than the PE method? The all-data method can be considered an affective exergame, and the all-data with PE comparison thus represents a direct comparison of affective and performance-based exergames. In our previous study, including personality and physiological measurements increased psychological state classification accuracy compared with using only performance measures [33], but this is not guaranteed to result in a better user experience. The inclusion of personality characteristics was considered separately because tailoring DDA to personality may improve user experience without additional sensors [34];
- Research question 2: do manual and random DDA result in the best and worst user experience, respectively? The user’s preference is commonly used as theground truth for training DDA algorithms in affective games [8,11,21], so manual DDA should result in a very positive user experience. At the same time, some studies have shown better user experience with physiology-based DDA than with manual DDA [25,26]. Random DDA should result in a poor experience but is included as a baseline;
- Research question 3: is user experience positively correlated with the accuracy of psychological state classification during gameplay? Our previous Wizard of Oz [29,30] studies and others’ studies [28] indicated a correlation between the two, but the nature of this relationship in actual games remains unclear.

## Methods

### Overview

This paper describes a comparative evaluation of five DDA methods in an affective exergame. Three of these methods were developed based on data recorded in a previous study [33]; thus, we briefly refer to a previous study for better understanding. Both studies were approved by the University of Wyoming Institutional Review Board (protocol no 20190822DN02495).

This section is divided into five subsections. The first subsection presents the exergame used for DDA evaluation, which is identical for both studies. The second subsection describes the different measurement types used as a basis for DDA, which were nearly identical for both studies. The third subsection summarizes the previous study used to train the DDA methods for this study (described in detail in our previous paper [33]). The fourth subsection presents the study protocol. Finally, the fifth subsection presents the outcome variables and data analysis performed to compare the five DDA methods in this study.

### Exergame

**Figure 1:**
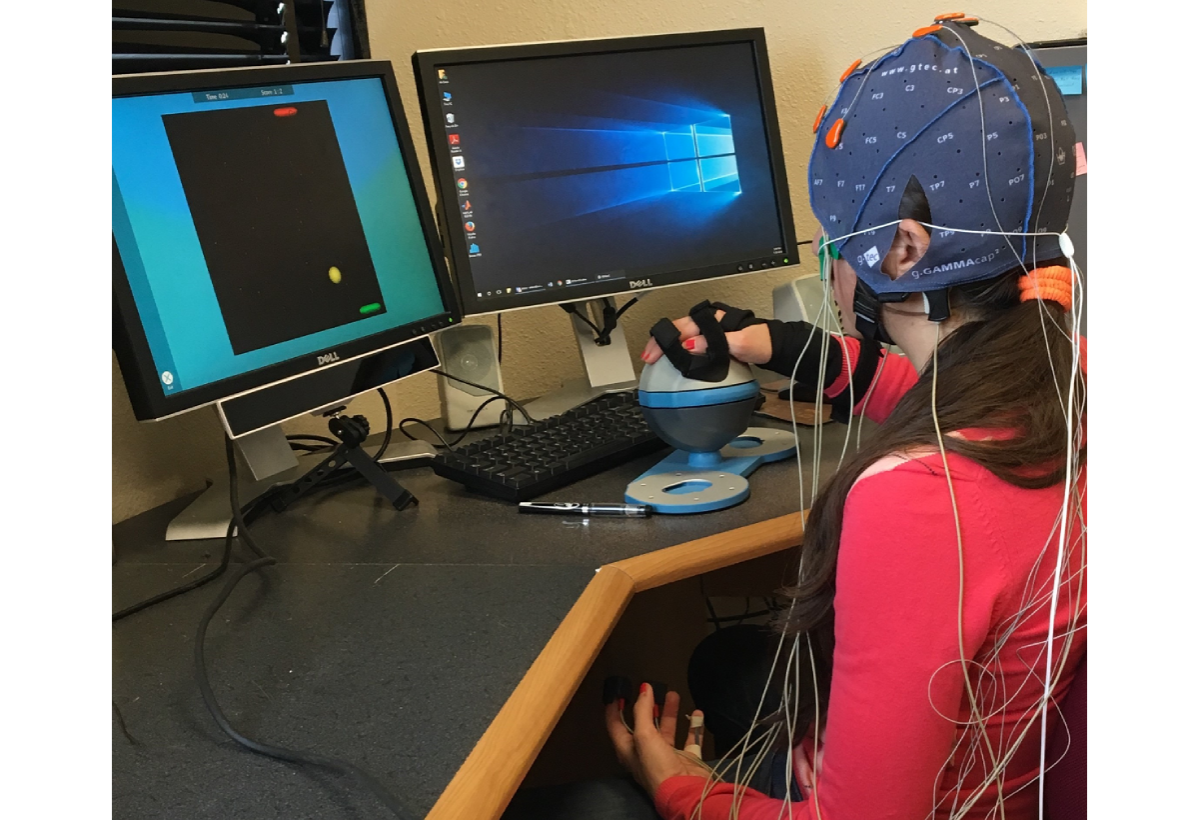
A participant playing the exergame (on the screen) using the Bimeo device (right hand) while wearing the different physiological sensors on the head and nondominant hand. The eye tracker is visible below the screen.

An exergame version of Pong was reused from our previous research, originally intended for two-player rehabilitation exergaming [35,36], and a single-player version was created for our previous open-loop study [33]. It consists of 2 paddles and a ball on a board. The participant controlled the bottom paddle, while the top paddle was controlled by a computer opponent. If the ball passed either player’s paddle, the other player would score a point, and the ball would start moving again from the middle of the screen. The player moved their paddle left and right by tilting the Bimeo (Kinestica) arm tracking device left and right with their dominant hand. The game was played on a 21-inch screen, with the participant seated approximately 60 cm from the screen. A photograph of this study setup is shown in Figure 1.

The game difficulty can be adjusted using two parameters: ball speed and paddle size. Although the game allows the participant’s and opponent’s paddle sizes to be changed independently, the evaluated DDA methods always changed the 2 paddles simultaneously and identically so that the participant’s paddle was never larger or smaller than that of the computer opponent. The DDA methods used in this study were based on multiple measurements, with the decision-making rules trained based on data recorded in a previous study, as described in the following sections.

### Measurements Used as the Basis for DDA

A total of 3 data types were recorded and used as a basis for DDA: physiological responses, performance, and personality characteristics. Physiological responses and performance were recorded continuously during gameplay and thus varied as the difficulty of the game changed. Personality characteristics were collected using questionnaires at the start of the session (after the researcher demonstrated the game but before participants played it) and did not change during gameplay but were expected to influence the participant’s DDA preferences.

The physiological recording process was identical in this study and our previous open-loop study [33] so that the previous data could be directly used to create the DDA methods; parts of the text in this section and Multimedia Appendix 1 are thus rephrased from the previous paper.

#### Physiological Signals

This study used 2 g.USBamp signal amplifiers and associated sensors (g.tec Medical Engineering GmbH) to measure six physiological signals: 8-channel EEG, 2-channel electrooculogram, ECG, respiration, galvanic skin response, and skin temperature. A seventh physiological signal, point of gaze, was recorded using a GP3 eye tracker (Gazepoint). The sensors used are shown in Figure 1. Detailed information about the measurements is available in Multimedia Appendix 1 but, in brief, they were performed as follows:

- EEG was recorded from 8 locations based on the 10-20 placement system [37]: AF3, AF4, F1, F2, F5, F6, C1, and C2. Feature extraction methods included the lateral power spectrum density [38] and dispersion entropy [39].
- Electrooculogram was recorded from 2 channels reflecting up-down and left-right eye movements. The extracted features were based on its first derivative. In addition, it was used to denoise the EEG signals.
- ECG was recorded using 4 electrodes on the trunk. Extracted features included heart rate and time- and frequency-domain estimates of heart rate variability [40].
- Respiration was recorded using a thermistor-based sensor in front of the nose and mouth. Extracted features included respiration rate and time-domain estimates of respiratory rate variability.
- The skin temperature was recorded using a sensor attached to the little finger of the nondominant hand. Extracted features included the mean temperature and changes in skin temperature across time.
- Galvanic skin response was recorded by attaching a sensor to the index and middle fingers of the nondominant hand. Features were extracted from both the tonic (low frequency) component and the phasic skin conductance responses [41].
- From the eye tracker data, the extracted features included the size of each pupil and the mean gaze velocity.

Physiological features were normalized by dividing each calculated feature value by the feature value obtained during the baseline period.

#### Performance

The in-game score was used as the only feature to assess participants’ performance. It was defined as the difference between the participant’s score and the computer opponent’s score.

#### Personality Characteristics

Participants completed four personality questionnaires: the Learning and Performance Goal Orientation measure [42], the Behavioral Inhibition and Activation Scales [43], the Self-Efficacy Scale [44], and the 10-Item Personality Inventory [45]. Questionnaire details are provided in Multimedia Appendix 1*.*

### Summary of the Previous Study

In our previous study [33], 30 healthy university students (mean 24.2, SD 4.4 years; 11 women) participated in a 1-hour session. Physiological signals were first recorded for a 2-minute baseline period, during which participants were instructed to relax, remain motionless, and look at the computer screen. Then, nine game difficulty configurations consisting of combinations of three possible ball speeds (slow, medium, and fast) and three possible paddle sizes (small, medium, and large) were played in random order for 2 minutes each. After each 2-minute interval, a short questionnaire was filled out to assess the perceived difficulty, enjoyment, and two subjective preferences about game difficulty (desired change to ball speed and paddle size). Participants had seven options for perceived difficulty and enjoyment (1 for very low and 7 for very high) and five options for how they would like to change each difficulty parameter, ranging from a decrease by two levels (−2) to an increase by two levels (+2). The order of game difficulty settings was preset randomly for each participant, and the participant’s desired changes to the ball speed and paddle size did not actually affect the game.

The study protocol resulted in a data set with two outputs (desired changes to ball speed and paddle size) and 64 input features calculated during 2-minute gameplay periods: the physiological, personality, and performance features described in Multimedia Appendix 1. Multiple classifiers were developed to categorize the two subjective difficulty adjustment preferences into three classes: increase (+1 and +2), no change (0), and decrease (−1 and −2). Separate classifiers were developed and evaluated for ball speed and paddle size, and separate classifiers were trained for different combinations of the three recorded data types (Multimedia Appendix 1). These classifiers were separated so that a classifier did not have access to the results of another classifier (eg, the ball speed classifier did not have access to the results of paddle size classification and vice versa); however, they were still trained on data from the same participants and gameplay periods, and all had access to current ball speed and current paddle size (as the current game state would be realistically available to any classifier). In all cases, stepwise forward feature selection [46] with an inclusion threshold of *P*=.05 was first used to select a subset of features. This reduced subset was then used to train four classifier types, of which multiple linear regression resulted in the highest classification accuracy and was thus chosen for this study.

### Study Protocol for Comparison of DDA Methods

This study compared five DDA methods using the same study setup and a protocol similar to the open-loop study. A total of 50 healthy university students (mean 25.1, SD 5.9 years; 13 women; 5 left-handed) participated in the study, with 10 assigned to each DDA method. The 5 participant groups went through the same study protocol, which differed only according to the DDA method they were assigned to. Participants were not told which method they were assigned to and had no way of identifying it.

**Figure 2:**
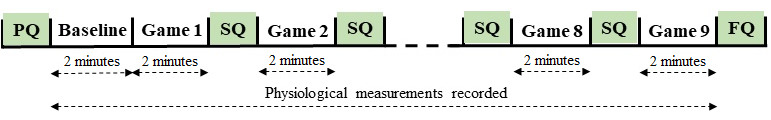
Summary of the study protocol. All participants first completed the personality questionnaires, rested for a 2-minute baseline period, then went through nine 2-minute gameplay intervals (game 1-9), with a short questionnaire after each. Physiological measurements were recorded throughout the baseline and gameplay and separated into 2-minute intervals for analysis. At the end, participants filled out the final outcome questionnaires. FQ: final questionnaire; PQ: personality questionnaire; SQ: short questionnaire.

After signing the consent form, filling out the four personality questionnaires, putting on the physiological sensors, and recording the responses for a 2-minute baseline period (same as in the *Summary of the Previous Study* section), participants started to play the game with a medium difficulty level (speed level 3 in a range of 1-6 and paddle size 2 in a range of 1-4). Every 2 minutes, the game was paused, and participants filled out a short questionnaire to report their perceived difficulty, enjoyment, and the way they would like to change the game parameters (same as in the *Summary of the Previous Study* section). Participants were allowed to ask for longer breaks in case of dizziness or arm fatigue; however, this only happened after a 2-minute interval among all 50 participants, and dizziness and fatigue were not otherwise tracked. Once the participant was ready to continue, the difficulty level was adjusted by changing the ball speed and paddle size using the DDA method to which the participant was assigned. In all DDA methods, the ball speed and paddle size were adjusted independently of each other. All participants played the game for a total of 18 minutes (nine 2-minute intervals, with difficulty adjustment after each interval) and then completed two outcome questionnaires (see the Outcome Variables and Data Analysis section) to complete the study protocol. The protocol is summarized in Figure 2.

As mentioned earlier, the participant groups differed according to the DDA method; in total, five methods were used:

1. Manual adjustment: the ball speed and paddle size were adjusted based on the participant’s preferences expressed in the short questionnaire. For each parameter, they had three options: increase by 1 level, no change, and decrease by 1 level.
2. Random adjustment: for both ball speed and paddle size, one of the three options available to the manual method (increase, no change, or decrease by one level) was chosen entirely randomly.
3. PE method: the ball speed and paddle size were adjusted based on three features: the current ball speed, current paddle size, and the in-game score achieved by participants. To perform the adjustment, two multiple linear regression models were trained based on data from a previous study using the three features as the inputs and the adjustment to the ball speed and paddle size as outputs (the exact regression coefficients are presented in Table A1 in Multimedia Appendix 1). The output of each regression model was converted to discrete classes and used to perform difficulty adjustment: increase by 1 level (output of model above 0.5), no change (output between −0.5 and 0.5), and decrease by 1 level (output below −0.5).
4. PEPE method: the ball speed and paddle size were adjusted based on multiple features selected among current game difficulty, in-game scores, and personality characteristics. The adjustment was performed using two regression-based classifiers, as with the PE method, but trained using both performance and personality data. A mix of personality characteristics from all four questionnaires was included in the classifiers, with only self-efficacy and agreeableness included in both classifiers (exact regression coefficients are presented in Tables A2 and A3 in Multimedia Appendix 1).
5. All-data method: the ball speed and paddle size were adjusted based on multiple features selected among current game difficulty, in-game score, personality characteristics (eg, extroversion), and physiological responses (eg, respiration rate). Adjustments were made using two regression-based classifiers developed based on data from a previous study. For both ball speed and paddle size classifiers, the first two selected features were from skin temperature and respiration, and multiple EEG features and multiple personality characteristics were selected (exact regression coefficients are presented in Tables A4 and A5 in Multimedia Appendix 1).

Among the five DDA methods, only the manual method took the participants’ preferences regarding game difficulty into account. Furthermore, limits were put in to ensure that the ball speed and paddle size did not exceed the preset limits. Specifically, if a DDA method’s decision would have caused a parameter to exceed a minimum or maximum value (1-6 for ball speed; 1-4 for paddle size), it instead stayed at that extreme value.

### Outcome Variables and Data Analysis

The primary outcome of the study was the effect of different DDA methods on user experience. In addition, two secondary analyses were performed. First, the closed-loop classification accuracy was calculated for the 50 participants and correlated with the user experience. Second, the classifiers were retrained on the data from the 50 participants using the same methods as in the previous study.

#### Effect of DDA Methods on User Experience

The effect of the different DDA methods on user experience was assessed using two self-report questionnaires at the end of the 18-minute gameplay period: the Intrinsic Motivation Inventory (IMI) [47] and the Flow Experience Measure (FEM) [48]. The IMI is an 8-item questionnaire that assesses effort/importance, perceived competence, interest/enjoyment, and pressure/tension with two items per assessed variable. The same version was used in our previous exergaming research [35,36]. The FEM assesses a single, variable flow, using 8 items. This resulted in 5 outcome variables in total, which were compared between the methods using two-tailed two-sample *t* tests. Intrinsic motivation and flow are perhaps the two most commonly evaluated short-term outcomes of serious games and exercise [6,19,23,25,44,47] and were thus considered appropriate for this study.

#### Accuracy of Speed and Paddle Size Changes

In this study, three DDA methods (PE, PEPE, and all-data) used regression-based classifiers developed based on data from a previous study [33]. Their accuracy was defined as the percentage of agreement between the classifier’s opinion and the participant’s preference for changes in ball speed or paddle size (measured via the short questionnaire after each 2-minute gameplay interval). This accuracy was expected to correlate with user experience when the classifier was used as a basis for difficulty adjustment. In a previous study, the all-data method resulted in the most accurate classification, whereas PE resulted in the least accurate classification; however, the accuracies in this study may be different for multiple reasons. For example, the range of ball speeds in this study was wider than in the previous study, and new situations, even within the same scenario, may induce ungeneralizable physiological responses, significantly reducing classification accuracy [27]. Simply having new participants may also reduce classification accuracy, although this was expected to have a smaller influence as participants in this and previous studies were drawn from the same broad pool. Thus, the classification accuracy was recalculated for each DDA method using data from this study.

To determine whether the accuracy of changes in ball speed and paddle size was correlated with user experience, Spearman correlation coefficients were calculated between each participant’s accuracy of ball speed and paddle size adjustments and the IMI and FEM outcomes. This was done across 40 participants; the manual group was dropped because they all had an accuracy of 100%.

#### Classifier Retraining and Validation

As mentioned in the previous subsection, we expected that the accuracy of the three classifiers used for the three DDA methods (PE, PEPE, and all-data) would not be the same in this study as it was in the previous study [33]. Although the data collection in this study was performed with prebuilt classifiers, these classifiers could be retrained offline after the data collection conclusion to determine whether the all-data method is still the most accurate if validated on the new data.

To perform this training and revalidation, three combinations (PE, PEPE, and all-data) of the three data types (physiology, performance, and personality characteristics) from 80 participants (30 from a previous study and 50 from this study) were used as inputs to train classifiers that categorize perceived difficulty, enjoyment, desired change to ball speed, and desired change to paddle size (obtained from the short questionnaire) into three classes: *low/decrease*, *medium/no change*, and *high/increase*. Reference output values for all classifiers were obtained from the short questionnaire, and Table 1 presents the selected reference ranges of the short questionnaire answers for each class and variable. These ranges were chosen to provide the most even possible spread of samples among the three classes for each variable (although an even spread was not always feasible because of biases in the data). The current ball speed and paddle size were added to all input data combinations because they indicate the current game state and are available to any practical model.

**Table 1:** Defined ranges for retraining the three-class classifiers.

Class		Difficulty	Enjoyment	Speed change	Paddle size change
**Low**					
	Range	1 to 2	1 to 4	−2 to −1	−2 to −1
	Sample, n	180	246	62	151
**Medium**					
	Range	3 to 5	5	0	0
	Sample, n	364	184	303	368
**High**					
	Range	6 to 7	6 to 7	1 to 2	1 to 2
	Sample, n	176	290	355	355

To reduce the number of features before classification, stepwise forward feature selection [46] with an inclusion threshold of 0.05 was used to find the most informative set of features. Then, to classify the input data, four different classifiers were used: a support vector machine with a linear kernel, linear discriminant analysis, ensemble decision tree, and multiple linear regression. The classifiers were validated using 10-fold cross-validation (72 participants’ data used to train and 8 participants’ data to validate the classifier; the procedure was repeated 10 times with each participant in the validation data set once).

## Results

### Effects of DDA Methods on User Experience

**Figure 3:**
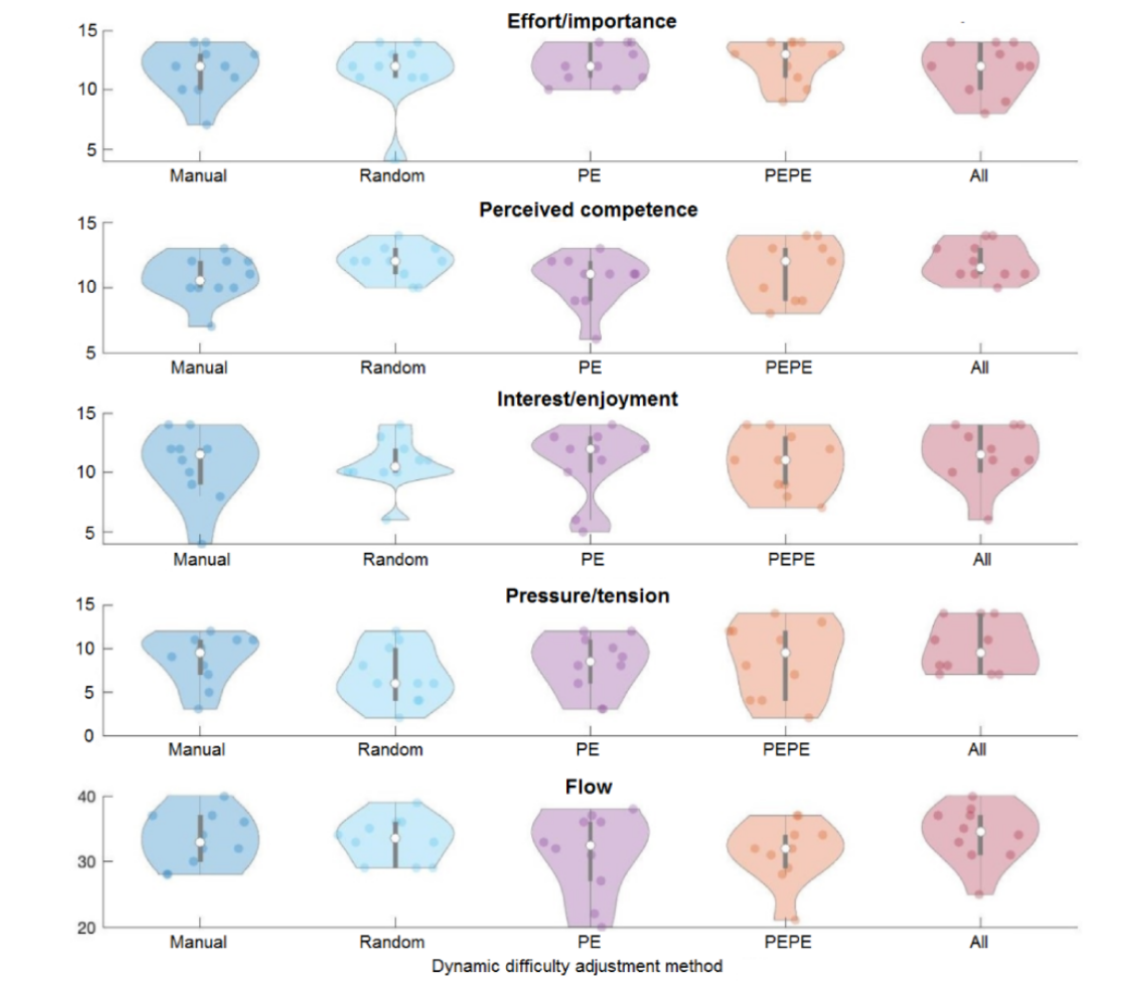
Violin plot of postexperiment self-report questionnaire outcomes. all data: based on physiology, personality, and performance. Each participant is indicated as a dot. The first four questionnaire outcomes have a possible range of 2-14, whereas flow has a possible range of 8-40. PE: adjustment based on performance; PEPE: adjustment based on personality and performance.

All extracted features and questionnaire results are available in Multimedia Appendix 2. Figure 3 shows the five outcomes of the IMI and FEM for all five DDA methods, presented as a violin plot of the 10 participants assigned to each method. The outcomes of the IMI and FEM were compared between different DDA methods using two-tailed two-sample *t* tests; however, only one difference was significant: pressure/tension was higher in the all-data method than in the random method (*P*=.04).

### Classifier Accuracy and Retraining

Table 2 shows the accuracy of each DDA method in the dynamic prediction of the participants’ desired changes to the game difficulty parameters. Accuracy was calculated by comparing the classifier’s prediction and the participants’ preference expressed on the short questionnaire after all 2-minute intervals. The manual method trivially achieves 100% accuracy because it uses the participants’ answers as a basis for difficulty adjustment. Among the other four methods, the all-data method was the most accurate for predicting the desired adjustments to ball speed and paddle size.

**Figure 4:**
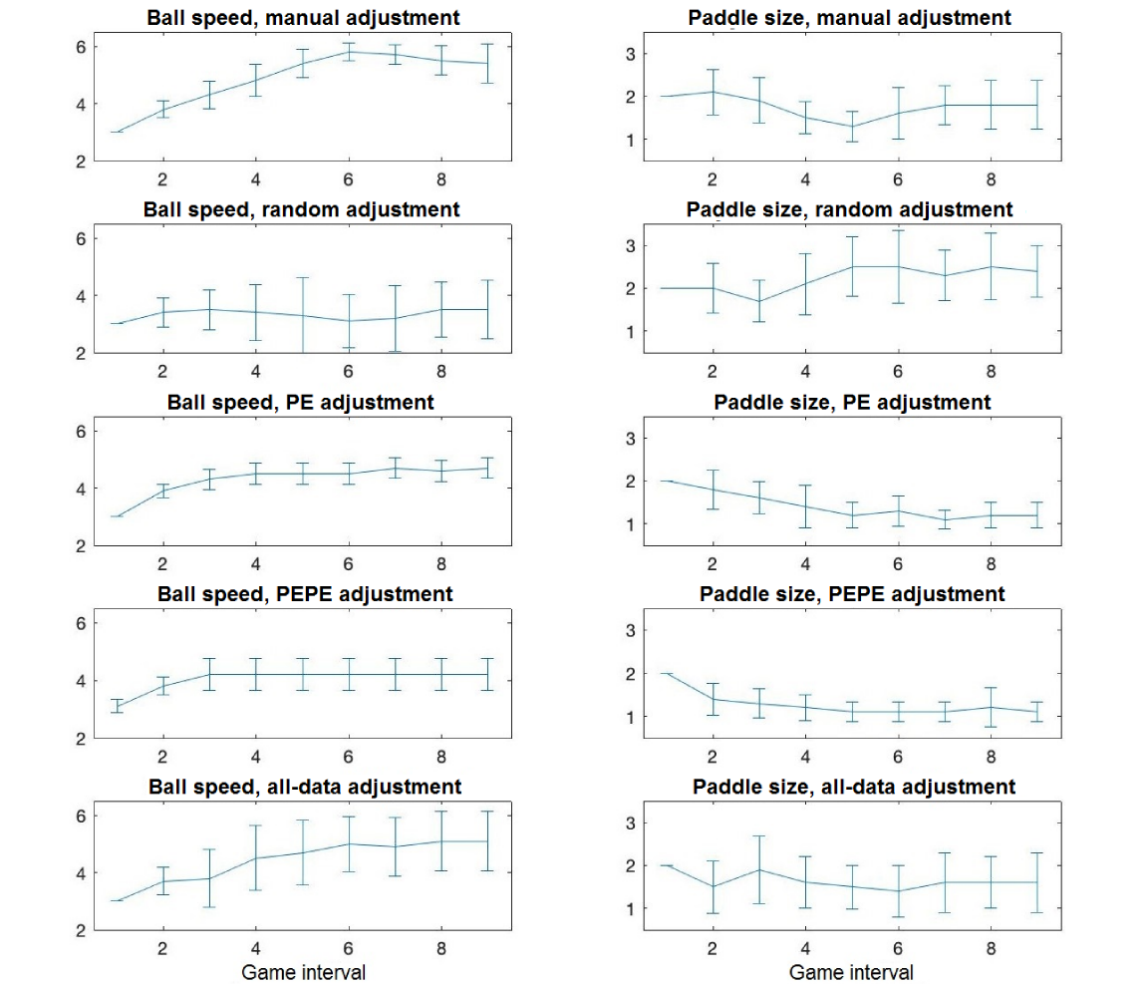
Ball speed (left) and paddle size (right) of five dynamic difficulty adjustment methods for nine 2-minute game intervals across 10 participants. Error bars indicate 95% CI. PE: based on performance; PEPE: based on personality and performance; all data: based on physiology, personality, and performance.

Figure 4 depicts the ball speed and paddle size for nine 2-minute intervals of each DDA method.

Table 3 presents the accuracies of three-class classification of the four answers to the short questionnaire when retraining and validating the classifiers using three combinations of input data types obtained from 80 participants. Combining all data types (all-data) resulted in the most accurate classifiers.

**Table 2:** Prediction accuracy of changes to the game difficulty parameters.

Difficulty parameter	Difficulty adjustment method (%)				
	Manual	Random	PE^a^	PEPE^b^	All-data^c^
Ball speed	100	32.5	53.7	43.7	62.5
Paddle size	100	41.2	46.2	33.5	55.0

^a^Based on performance.

^b^Based on personality and performance.

^c^Based on physiology, personality, and performance.

**Table 3:** Mean three-class classification accuracies for different combinations of input data modalities.

Input data	Outcome variable (%)			
	Difficulty	Enjoyment	Speed change	Paddle size change
PE^a^	62.4 (S^b^)	50.2 (L^c^)	64.6 (E^d^)	55.8 (E)
PEPE^e^	62.5 (S)	50.7 (S)	62.9 (S)	59.9 (R^f^)
All-data	64.2 (S)	50.7 (S)	62.6 (E)	61.7 (R)

^a^Based on performance.

^b^S: support vector machine.

^c^L: linear discriminant analysis.

^d^E: ensemble decision tree.

^e^Based on personality and performance.

^f^R: multiple linear regression.

### Correlation Between Classifier Accuracy and User Experience

Finally, Table 4 presents the Spearman correlation coefficients of the outcomes of the IMI and FEM questionnaires and the accuracy of ball speed and paddle size adjustments across all DDA methods except manual (where it was always 100%).

**Table 4:** Spearman correlation coefficients between user experience indicators and accuracy of difficulty adjustments.

User experience aspect	Difficulty parameter			
	Ball speed		Paddle size	
	Coefficient	*P* value	Coefficient	*P* value
Effort/importance	0.27	.09	−0.14	.37
Enjoyment/interest	0.38	.01	−0.10	.51
Competence	0.16	.31	−0.03	.84
Pressure/tension	0.43	.005	−0.04	.81
Flow	0.30	.06	−0.05	.77

## Discussion

### Practical Implications

No differences were observed between the DDA methods. The exception was higher pressure/tension with the all-data method than with the random method, which was expected as the all-data method resulted in greater difficulty (Figure 4); similar higher pressure/tension as a result of adaptation versus no adaptation has been observed in our previous work [35]. Although we shall discuss the limitations of this study later, we first discuss the practical implications of the results.

As there was no clear benefit to the all-data method, our results do not support the use of physiological measurements for affective exergaming in practical environments. Physiological sensors are expensive, time-consuming, and inconvenient; thus, they should not be used unless they show a clear benefit. This result disagrees with the study of Xu et al [14], which found positive effects of physiology-based DDA, but it is difficult to compare the 2 studies because of significant methodological differences; for example, the Xu study had only three difficulty levels in total. At first, the result also appears to disagree with a previous study by Liu et al [8], which found positive effects of physiology-based DDA in an entertainment game. However, Liu et al [8] also did not find significant differences between physiology-based DDA and performance-based DDA based on outcome measures obtained at the end of the session; they were only able to show a difference because outcome measures were also obtained at multiple time points during gameplay itself, and those midgame outcomes were significant.

The prediction accuracy of changes to game difficulty parameters was higher with the all-data method than all other automated methods (Table 2), so the inclusion of physiological measurements did increase accuracy. For example, the accuracies for ball speed and paddle size were 62.5% and 55.0% with the all-data method compared with 53.7% and 46.2% with the PE method, respectively. The difference in accuracy may have been too low to improve the user experience meaningfully. Our previous Wizard of Oz study, for example, found that users did not reliably perceive a difference between psychological state classification accuracies that differed by less than 10% [30]. Thus, other games where adding physiological measurements increased DDA accuracy by less than 10% (including our own previous work [12] and the work of Chanel et al [9]) also may not find an improvement in user experience as a result.

As seen in Table 4, the accuracy of DDA correlated with user enjoyment/interest (*r*=0.38; *P*=.01) when the four nonmanual DDA groups were pooled together. This result agrees with our previous research [29,30] and with studies from other authors [28]. Thus, our study supports the value of more effective, personalized DDA methods; it simply does not support the premise that physiology-based DDA will be more effective than performance-based DDA.

Finally, a follow-up analysis was conducted to identify any clear patterns in when and how the DDA methods made mistakes. A clear pattern was observed: all DDA methods were more accurate at extreme difficulties (eg, very high/low paddle sizes) than at moderate difficulties. This is, in our opinion, unsurprising: when the difficulty is set to an extreme level, the correct choice is very likely to be moving away from the extreme. However, when difficulty is moderate, the correct choice is more dependent on the individual player.

### Differences in Classifier Performance

Three DDA methods used classifiers that were trained on data from a previous open-loop study [33]. Studies from other fields of human-machine interaction have shown that classifiers that perform well in an offline, open-loop setting may not perform equally well in an online, real-time setting where they are used to influence machine behavior [31,32]. Thus, although our primary research focus was on user experience, we were also interested in whether the previously trained classifiers would exhibit lower accuracy in this study. Indeed, the accuracies in this study (Table 3) were lower than those in the previous one. For example, in the previous study, the all-data method resulted in a classification accuracy of 84.1% for both ball speed and paddle size preferences [33]; in this study, the accuracies were 62.5% for ball speed and 55% for paddle size. As a validation step, we reran the classification offline (post hoc) to ensure there was no bug with real-time processing; this resulted in the same results as during the data collection sessions.

We believe that the primary reason for the decrease in classification accuracy is the difference in the experienced situations. In a previous study [33], participants were exposed to three different ball speeds and three paddle sizes. In this study, participants were exposed to six different ball speeds and four paddle sizes. This is partially a weakness of our study design; however, in realistic applications, psychological state classification algorithms would inevitably be exposed to situations not seen in the training data. As a follow-up analysis, we retrained and evaluated the same types of classifiers using data from all 50 participants in this study and the 30 participants from the previous one (80 participants in total). As shown in Table 3, this also resulted in lower accuracies than those in the previous study [33], and we again believe that the primary reason for the difference was the broader range of experienced situations.

Nonetheless, as another practical implication, this result suggests that psychological state classification accuracies obtained during offline classifier training on previously recorded data may not transfer to a real-time DDA context. Thus, although a higher DDA accuracy correlates with user experience (Table 4), any exergame studies that only show differences in offline classification accuracy (including our own previous work [33]) should be taken with a grain of salt because these offline results are not guaranteed to have practical benefits when applied to DDA.

### Could Other Study Designs Be More Sensitive?

As our study did not show differences in user experience among the DDA methods, we must ask whether other study designs would be more sensitive to these differences. Indeed, an intuitive follow-up study would be to test the same DDA methods in a within-subjects design, with each participant experiencing multiple DDA methods. Xu et al [14] and Liu et al [8] found differences in user experience between physiology-based and performance-based DDA, both of which used a within-subjects design, similar to our previous Wizard of Oz study [30]. In this study, we opted for a between-subjects design so that participants could experience their assigned DDA method for a longer amount of time; using multiple DDA methods would have resulted in much longer sessions. However, we acknowledge that this is not necessarily optimal.

Instead of a within-subjects design, it would also be possible to increase the sample size. The 10 participants assigned to each DDA method in this study constitute a relatively small sample size that may have been insufficient to find differences even if they did exist. We originally envisioned a larger sample size; however, data collection was carried out in early 2020 and then interrupted by the COVID-19 pandemic, forcing us to limit ourselves to the collected 50 participants.

Finally, in addition to a within-subjects design and/or a larger sample size, additional qualitative data could have been collected. For example, players could have been asked open-ended questions about their experiences. This data collection was done in the study by Liu et al [8], where participants reported lower anxiety with physiology-based DDA in open-ended questions but did not report lower anxiety on postgame forced-choice questionnaires. Although no systematic qualitative data collection was performed in this study, we mention an experimenter observation: although participants did not know which DDA method they had been assigned to, some appeared to have initial biases (eg, whether useful information can be extracted from physiology), and some attempted to determine whether their physiology influenced the system by, for example, breathing rapidly. This is in line with previous publications about participant bias and bidirectional relationships in affective computing [27,28] and may warrant further investigation.

### Limitations of the Used Exergame

Finally, the results of our study do not necessarily generalize to all affective exergames. We believe that the choice of three-class classification followed by increasing or decreasing or not changing difficulty is not controversial, as it represents a classic approach to affective gaming [21]. However, our study adjusted two difficulty parameters (ball speed and paddle size) simultaneously, and the results may not be generalizable to games where only a single difficulty parameter is changed, such as in the work of Liu et al [8]. Furthermore, our study changed difficulty in relatively small steps, and our results may not generalize to games where a single DDA decision may immediately change the difficulty to a very high or very low level, such as in the work of Xu et al [14].

In addition, our study is based on the classic affective gaming approach of classifying difficulty based on prerecorded data and then adapting it at a constant frequency without user input [21]. Thus, it may not generalize to designs where DDA is adapted with a variable frequency, although such designs are not yet common in affective computing; it also may not generalize to designs where users can provide feedback about DDA decisions, potentially increasing future accuracy. For example, reinforcement learning, which remains largely unexplored in affective exergames, could be used for this purpose—users could give *rewards* for correct DDA decisions, gradually leading to a more positive user experience. Furthermore, the results may not generalize to designs that omit classification entirely and use other schemes, such as fuzzy control [5] or random forest–based regression [16].

Finally, different results might be obtained in the same scenario using different data analysis methods. For example, improved physiological feature extraction and classification may increase the accuracy of physiology-based DDA, resulting in an improved user experience in the same setting; conversely, improved performance feature extraction may also increase the accuracy of performance-based DDA. If we were to reuse the data for further research, we would likely first investigate alternatives to stepwise feature selection, which has potential issues and may not select the optimal subset of features [49]. As a follow-up analysis, we retrained and evaluated the same classifiers with two different feature reduction methods (principal component analysis and the lasso method, recommended by an article critiquing stepwise methods [49]) but did not find an improvement in classification accuracy as a result of these methods.

### Conclusions

Five aspects of user experience were compared among five DDA methods of a Pong exergame: manual, random, PE, PEPE, and all-data. The last three methods adjusted the ball speed and paddle size in the game using regression-based classifiers developed in a previous (open-loop) study with 30 participants.

Though the all-data method exhibited the highest user state classification accuracy (approximately 10% higher than the PE method), no significant differences in user experience were observed among DDA methods. Thus, our results do not support the addition of physiological measurements to affective exergames; as such measurements are expensive and time-consuming, they should only be added if they meaningfully improve user experience. The study found that user experience was correlated with user state classification accuracy, thus supporting the development of more effective DDA methods, which simply does not support the notion that additional measurements will automatically improve the user experience.

The study results are limited by a somewhat suboptimal study design: each participant experienced only one DDA method, and a within-subjects design in which participants experience multiple methods may find positive effects of more complex DDA methods on user experience. Nonetheless, few studies have examined the relative effects of physiology-based DDA on user experience, and our study thus adds to the limited body of evidence on the effects of physiological measurements in affective games on user experience.

## Appendix

Multimedia Appendix 1 Details about physiological measurements, questionnaires, and linear regression–based models.

Multimedia Appendix 2 Results from individual participants in each game condition.

## Abbreviations

DDA: dynamic difficulty adjustment
ECG: electrocardiogram
EEG: electroencephalography
FEM: Flow Experience Measure
IMI: Intrinsic Motivation Inventory
PE: performance based
PEPE: personality-performance based

## References

[1] Ko J, Jang S, Lee HT, Yun H, Kim YS (2020). Effects of virtual reality and non-virtual reality exercises on the exercise capacity and concentration of users in a Ski exergame: comparative study. JMIR Serious Games.

[2] O'Loughlin EK, Barnett TA, McGrath JJ, Consalvo M, Kakinami L (2019). Factors associated with sustained exergaming: longitudinal investigation. JMIR Serious Games.

[3] Brauner P, Ziefle M (2020). Serious motion-based exercise games for older adults: evaluation of usability, performance, and pain mitigation. JMIR Serious Games.

[4] Laver KE, George S, Thomas S, Deutsch JE, Crotty M (2015). Virtual reality for stroke rehabilitation. Cochrane Database Syst Rev.

[5] Rodriguez-Guerrero C, Knaepen K, Fraile-Marinero JC, Perez-Turiel J, Gonzalez-de-Garibay V, Lefeber D (2017). Improving challenge/skill ratio in a multimodal interface by simultaneously adapting game difficulty and haptic assistance through psychophysiological and performance feedback. Front Neurosci.

[6] Chen J (2007). Flow in games (and everything else). Commun ACM.

[7] Silva MP, Silva VD, Chaimowicz L (2017). Dynamic difficulty adjustment on MOBA games. Entertain Comput.

[8] Liu C, Agrawal P, Sarkar N, Chen S (2009). Dynamic difficulty adjustment in computer games through real-time anxiety-based affective feedback. Int J Hum-comput Int.

[9] Chanel G, Rebetez C, Bétrancourt M, Pun T (2011). Emotion assessment from physiological signals for adaptation of game difficulty. IEEE Trans Syst Man Cybern, Part A Syst Humans.

[10] Rani P, Sarkar N, Smith CA, Kirby LD (2004). Anxiety detecting robotic system – towards implicit human-robot collaboration. Robotica.

[11] Ng Y, Khong C, Thwaites H (2012). A review of affective design towards video games. Procedia Soc Behav Sci.

[12] Novak D, Mihelj M, Ziherl J, Olenšek A, Munih M (2011). Psychophysiological measurements in a biocooperative feedback loop for upper extremity rehabilitation. IEEE Trans Neural Syst Rehabil Eng.

[13] Shirzad N, Van der Loos HF (2016). Evaluating the user experience of exercising reaching motions with a robot that predicts desired movement difficulty. J Mot Behav.

[14] Xu G, Gao X, Pan L, Chen S, Wang Q, Zhu B, Li J (2018). Anxiety detection and training task adaptation in robot-assisted active stroke rehabilitation. Int J Adv Robot Syst.

[15] Ozkul F, Barkana DE, Masazade E (2021). Dynamic difficulty level adjustment based on score and physiological signal feedback in the robot-assisted rehabilitation system, RehabRoby. IEEE Robot Autom Lett.

[16] Novak D, Beyeler B, Omlin X, Riener R (2015). Workload estimation in physical human–robot interaction using physiological measurements. Interact Comput.

[17] Koenig A, Novak D, Omlin X, Pulfer M, Perreault E, Zimmerli L, Mihelj M, Riener R (2011). Real-time closed-loop control of cognitive load in neurological patients during robot-assisted gait training. IEEE Trans Neural Syst Rehabil Eng.

[18] Knaepen K, Marusic U, Crea S, Guerrero CD, Vitiello N, Pattyn N, Mairesse O, Lefeber D, Meeusen R (2015). Psychophysiological response to cognitive workload during symmetrical, asymmetrical and dual-task walking. Hum Mov Sci.

[19] Leiker AM, Miller M, Brewer L, Nelson M, Siow M, Lohse K (2016). The relationship between engagement and neurophysiological measures of attention in motion-controlled video games: a randomized controlled trial. JMIR Serious Games.

[20] Barathi S, Proulx M, O'Neill E, Lutteroth C (2020). Affect recognition using psychophysiological correlates in high intensity VR exergaming. Proceedings of the 2020 CHI Conference on Human Factors in Computing Systems.

[21] Novak D, Mihelj M, Munih M (2012). A survey of methods for data fusion and system adaptation using autonomic nervous system responses in physiological computing. Interact Comput.

[22] Bian D, Wade J, Swanson A, Weitlauf A, Warren Z, Sarkar N (2019). Design of a physiology-based adaptive virtual reality driving platform for individuals with ASD. ACM Trans Access Comput.

[23] Bontchev B (2016). Adaptation in affective video games: a literature review. Cybern Inf Technol.

[24] Jerčić P, Sundstedt V (2019). Practicing emotion-regulation through biofeedback on the decision-making performance in the context of serious games: a systematic review. Entertain Comput.

[25] Ewing KC, Fairclough SH, Gilleade K (2016). Evaluation of an adaptive game that uses EEG measures validated during the design process as inputs to a biocybernetic loop. Front Hum Neurosci.

[26] Nacke L, Kalyn M, Lough C, Mandryk R (2011). Biofeedback game design: using direct and indirect physiological control to enhance game interaction. Proceedings of the SIGCHI Conference on Human Factors in Computing Systems.

[27] Fairclough SH, Lotte F (2020). Grand challenges in neurotechnology and system neuroergonomics. Front Neuroergono.

[28] Fairclough S, Karran A, Gilleade K (2015). Classification accuracy from the perspective of the user: real-time interaction with physiological computing. Proceedings of the 33rd Annual ACM Conference on Human Factors in Computing Systems.

[29] Novak D, Nagle A, Riener R (2014). Linking recognition accuracy and user experience in an affective feedback loop. IEEE Trans Affect Comput.

[30] McCrea S, Geršak G, Novak D (2017). Absolute and relative user perception of classification accuracy in an affective video game. Interact Comput.

[31] Chase SM, Schwartz AB, Kass RE (2009). Bias, optimal linear estimation, and the differences between open-loop simulation and closed-loop performance of spiking-based brain-computer interface algorithms. Neural Netw.

[32] Hargrove LJ, Scheme EJ, Englehart KB, Hudgins BS (2010). Multiple binary classifications via linear discriminant analysis for improved controllability of a powered prosthesis. IEEE Trans Neural Syst Rehabil Eng.

[33] Darzi A, Wondra T, McCrea S, Novak D (2019). Classification of multiple psychological dimensions in computer game players using physiology, performance, and personality characteristics. Front Neurosci.

[34] Nagle A, Wolf P, Riener R (2016). Towards a system of customized video game mechanics based on player personality: relating the Big Five personality traits with difficulty adaptation in a first-person shooter game. Entertain Comput.

[35] Goršič M, M A, Novak D (2017). Comparison of two difficulty adaptation strategies for competitive arm rehabilitation exercises. Proceedings of the International Conference on Rehabilitation Robotics (ICORR).

[36] Gorsic M, Clapp JD, Darzi A, Novak D (2019). A brief measure of interpersonal interaction for 2-player serious games: questionnaire validation. JMIR Serious Games.

[37] Klem GH, Lüders H O, Jasper HH, Elger C (1999). The ten-twenty electrode system of the International Federation. The International Federation of Clinical Neurophysiology. Electroencephalogr Clin Neurophysiol Suppl.

[38] Fitzgibbon S, DeLosAngeles D, Lewis T, Powers D, Grummett T, Whitham E, Ward L, Willoughby J, Pope K (2016). Automatic determination of EMG-contaminated components and validation of independent component analysis using EEG during pharmacologic paralysis. Clin Neurophysiol.

[39] Azami H, Rostaghi M, Abásolo D, Escudero J (2017). Refined composite multiscale dispersion entropy and its application to biomedical signals. IEEE Trans Biomed Eng.

[40] Task Force of the European Society of Cardiology and the North American Society of Pacing and Electrophysiology (1996). Heart rate variability: standards of measurement, physiological interpretation, and clinical use. Eur Heart J.

[41] Boucsein W (2012). Electrodermal Activity.

[42] Kim TT, Lee G (2013). Hospitality employee knowledge-sharing behaviors in the relationship between goal orientations and service innovative behavior. Int J Hosp Manag.

[43] Carver CS, White TL (1994). Behavioral inhibition, behavioral activation, and affective responses to impending reward and punishment: the BIS/BAS scales. J Pers Soc Psychol.

[44] Hsia L, Huang I, Hwang G (2016). Effects of different online peer-feedback approaches on students' performance skills, motivation and self-efficacy in a dance course. Comput Educ.

[45] Gosling SD, Rentfrow PJ, Swann WB (2003). A very brief measure of the Big-Five personality domains. J Res Pers.

[46] Quinn GP, Keough MJ (2002). Multiple and complex regression. Experimental Design and Data Analysis for Biologists.

[47] Markland D, Hardy L (1997). On the factorial and construct validity of the Intrinsic Motivation Inventory: conceptual and operational concerns. Res Q Exerc Sport.

[48] Sung H, Hwang G, Yen Y (2015). Development of a contextual decision-making game for improving students' learning performance in a health education course. Comput Educ.

[49] Flom P, Cassell D (2007). Stopping stepwise: Why stepwise and similar selection methods are bad, and what you should use. Proceedings of the NESUG Conference.

