# Supplementary material for: User Experience With Dynamic Difficulty Adjustment Methods for an Affective Exergame: Comparative Laboratory-Based Study
Source: JMIR Serious Games. 2021 May 31;9(2):e25771. doi: 10.2196/25771 (PMC8204235; doi:10.2196/25771)
Supplement: Multimedia Appendix 1 [file games_v9i2e25771_app1.doc]

# Multimedia Appendix 1 for “User Experience with Dynamic Difficulty Adjustment Methods for an Affective Exergame: Comparative Lab-Based Study”

This appendix is divided into two parts. The first part describes all the features extracted from the physiological measurements and personality questionnaires. The second part describes the detailed linear-regression-based models used to adjust ball speed and paddle size in three dynamic difficulty adjustment methods.

## Details about measurements and extracted features

### Physiological measurements

As mentioned in the main text, seven physiological signals were recorded: 8-channel electroencephalogram (EEG), electrooculogram (EOG), electrocardiogram (ECG), respiration, galvanic skin response (GSR), skin temperature (ST), and point of gaze. The first six signals were all recorded using two g.USBamp signal amplifiers and associated sensors (g.tec Medical Engineering GmbH, Austria).

- EEG signals were recorded using the g.Sahara sensor from prefrontal, frontal and central areas of the brain based on the 10-20 placement system [1]: AF3, AF4, F1, F2, F5, F6, C1, and C2. The g.Sahara reference electrode was Cz and the ground electrode was placed on the right mastoid. While the manufacturer generally recommends placing both the reference and ground on the mastoids, reference placement at Cz is common in affective computing and also permitted by the manufacturer.
- 2-channel EOG (reflecting up-down and left-right eye movement) was recorded using small ECG electrodes placed around the eyes following suggestions in the literature [2]. The 2-channel EOG was used both as a source of information about psychological states and as a reference signal with which to denoise the EEG signals (which are influenced by eye activity). EEG denoising was done with a recursive least squares adaptive filter with EOG as the reference [3].
- ECG was recorded using four electrodes on the body (two on the chest, one over the spine, one on the abdomen) as recommended by the g.USBamp manufacturer.
- Respiration was recorded using a thermistor-based sensor in front of the nose and mouth.
- ST was recorded using a sensor attached to the distal phalanx of the little finger of the non-dominant hand.
- GSR was recorded using two electrodes (g.GSRsensor2) attached to the distal phalanges of the index and middle fingers of the nondominant hand.
- Point of gaze was recorded using a GP3 eye tracker (Gazepoint, Canada). The eye tracker was calibrated at the start of the session by having participants look at five points on the screen (one in each corner, one in center) for approximately 2.5 seconds per point. After calibration,

The sampling frequency was 30 Hz for point of gaze and 256 Hz for all other signals. Respiration, GSR, and ST were filtered with a 0-30 Hz bandpass filter to reduce high-frequency noise. ECG was filtered with a 5-Hz highpass filter to eliminate low-frequency noise, and a 60-Hz notch filter was used to remove electrical interference. EEG was filtered with a 2-60 Hz bandpass filter.

A total of 49 features were extracted from the physiological signals over each analysis interval (2 minutes) as follows:

- *EEG*: Two methods were used: lateral power spectrum density (PSD) [4] and dispersion entropy [5]. Lateral PSD resulted in 20 features (four bipolar signals times five frequency bands) while dispersion entropy resulted in eight features (one feature per unipolar signal). Of the five frequency bands for lateral PSD, one covered the alpha band (8-14 Hz), two covered the beta band (15-30 Hz), and two covered the gamma band (30-50 Hz).
- *EOG*: Mean, median and standard deviation of the EOG first derivative.
- *ECG*: Two time-domain features were calculated: mean heart rate and the standard deviation of inter-beat intervals. Furthermore, three frequency-domain features of heart rate variability were calculated: power of low frequencies (LF – 0.04-0.15 Hz), power of high frequencies (HF – 0.15-0.4 Hz) and the power ratio of LF/HF [6].
- *Respiration*: The mean respiration rate (number of complete breathing cycles per minute), the standard deviation of respiration rate, and the root-mean-square of successive differences of respiration periods.
- *ST*: Mean ST and the difference in ST between the first and last second of the analysis interval.
- *GSR*: The GSR is divided into two components: tonic (low-frequency) and phasic (high-frequency). For the tonic component, the mean GSR and the difference in GSR between the first and last second of the scenario were calculated. The phasic component consists of discrete skin conductance responses, and we calculated the number of responses, the mean response amplitude, and the standard deviation of response amplitude [7].
- *Eye tracker*: The size of each pupil (left and right separately) and mean gaze velocity based on the point of gaze estimated by the eye tracker’s built-in software [8].

### Personality questionnaires

Participants filled out four personality questionnaires after seeing the game demonstrated by the researcher, but before playing the game themselves.

- The Learning and Performance Goal Orientation measure [9]. The variant used in this study has 16 total items that measure learning goal orientation (8 items) and performance goal orientation (8 items). All items are seven-point Likert scales, with 1 representing “strongly disagree” and 7 representing “strongly agree”.
- The Behavioral Inhibition/Activation Scales [10] measure has 20 total items that measure behavioral inhibition (7 items), activation system reward responsiveness (5 items), activation system drive (4 items), and activation system fun seeking (4 items). All items are 4-point Likert scales, with 1 representing “strongly agree” and 4 representing “strongly disagree”, with no neutral option.
- A Self-Efficacy scale with 4 total items, adapted from Hsia et al. [11]. The four items are “I believe I will receive an excellent score in this game”, “I am certain I can master the skills needed for this game”, “I am confident I can perform the basic actions needed for this game”, and “I expect to do well in this game”. All items are 5-point Likert scales, with 1 representing “strongly disagree” and 5 representing “strongly agree”.
- The Ten Item Personality Inventory [12] measure has 10 total items that measure the Big Five personality characteristics with 2 items each: extraversion, agreeableness, conscientiousness, neuroticism, and openness to experiences. All items are 7-point Likert scales, with 1 representing “not at all true” and 7 representing “completely true”.

## Linear-regression-based models used as basis for dynamic difficulty adjustment

Table A.1 shows the selected features and regression coefficients for performance-based ball speed and paddle size adjustment models. Table A.2 and A.3 show the selected features and regression coefficients for personality-performance-based ball speed and paddle size adjustment models. Finally, Tables A.4 and A.5 show the selected features and regression coefficients for physiology-personality-performance-based ball speed and paddle size adjustment models.

Table A.1. Performance-based linear regression models used to adjust ball speed and paddle size. Both models are presented in the same table and include three features (score, current speed, current paddle size) and a constant term.

| **Model for** | ***Constant*** | ***In-game Score*** | ***Current Speed*** | ***Current Paddle Size*** |
| --- | --- | --- | --- | --- |
| Speed Change | 1.53 | 0.0400 | -0.4032 | 0.8023 |
| Paddle Size Change | 1.29 | -0.0900 | -0.0498 | -0.5850 |

Table A.2. Personality-performance-based linear regression model used to adjust ball speed.

| ***#*** | ***Feature*** | ***Coefficient*** | ***#*** | ***Feature*** | ***Coefficient*** |
| --- | --- | --- | --- | --- | --- |
| 1 | Constant | 0.9744 | 6 | Openness to Experiences | -0.0390 |
| 2 | Learning Goal | -0.0127 | 7 | Score | 0.0100 |
| 3 | Self Efficacy | 0.0552 | 8 | Current Speed | -0.4060 |
| 4 | Agreeableness | 0.0506 | 9 | Current Paddle Size | 0.0705 |
| 5 | Conscientiousness | 0.0464 |  |  |  |

Table A.3. Personality-performance-based linear regression model used to adjust paddle size.

| ***#*** | ***Feature*** | ***Coefficient*** | ***#*** | ***Feature*** | ***Coefficient*** |
| --- | --- | --- | --- | --- | --- |
| 1 | Constant | 1.1012 | 5 | Agreeableness | -0.0758 |
| 2 | Behavioral Inhibition | 0.0406 | 6 | Score | -0.0700 |
| 3 | Fun Seeking | 0.0613 | 7 | Current Paddle size | -0.5811 |
| 4 | Self Efficacy | -0.0609 |  |  |  |

Table A.4. Physiology-personality-performance-based linear regression model used to adjust ball speed. GSR = Galvanic Skin Response, PSD = Power Spectrum Density.

| ***#*** | ***Feature*** | ***Coefficient*** | ***#*** | ***Feature*** | ***Coefficient*** |
| --- | --- | --- | --- | --- | --- |
| 1 | Constant | 5.0994 | 9 | Dispersion Entropy, C1 | -2.5370 |
| 2 | Difference of Skin temperature | -2.9143 | 10 | Self Efficacy | 0.0682 |
| 3 | Std of respiration period | 0.1495 | 11 | Agreeableness | 0.0359 |
| 4 | Std of GSR phasic | -0.0044 | 12 | Conscientiousness | 0.0500 |
| 5 | Lateral PSD, AF3-4, Alpha 2 | -0.0023 | 13 | Score | 0.0056 |
| 6 | Lateral PSD, F1-2, Gamma | 0.0004 | 14 | Current Speed | -0.4101 |
| 7 | Lateral PSD, F5-6, Gamma | -0.0218 | 15 | Current Paddle size | 0.0796 |
| 8 | Lateral PSD, C1-2, Alpha 1 | -0.0126 |  |  |  |

Table A.5. Physiology-personality-performance-based linear regression model used to adjust paddle size. EOG = Electrooculogram, PSD = Power Spectrum Density.

| *#* | ***Feature*** | ***Coefficient*** | ***#*** | ***Feature*** | ***Coefficient*** |
| --- | --- | --- | --- | --- | --- |
| 1 | Constant | -2.1552 | 11 | Dispersion Entropy, F6 | 2.3482 |
| 2 | Difference of Skin temperature | 2.6755 | 12 | Dispersion Entropy, C1 | -2.3251 |
| 3 | Std of respiration period | -0.1446 | 13 | Behavioral Inhibition | 0.0191 |
| 4 | Std (abs (Gradient)) of EOG | 0.2240 | 14 | Fun Seeking | 0.0297 |
| 5 | Mean Gaze Velocity | -0.1657 | 15 | Self-Efficacy | -0.0437 |
| 6 | Lateral PSD, AF3-4, Beta 2 | -0.0043 | 16 | Extraversion | 0.0303 |
| 7 | Lateral PSD, F1-2, Alpha 1 | -0.0047 | 17 | Agreeableness | -0.0666 |
| 8 | Lateral PSD, F1-2, Beta 1 | -0.0027 | 18 | Score | -0.0055 |
| 9 | Dispersion Entropy, F2 | 5.6042 | 19 | Current Paddle size | -0.5648 |
| 10 | Dispersion Entropy, F5 | -4.7899 |  |  | |

## Appendix references

1. Klem GH, Lüders HO, Jasper HH, Elger C. The ten-twenty electrode system of the International Federation. The International Federation of Clinical Neurophysiology. Electroencephalogr Clin Neurophysiol 1999;52:3–6. PMID:10590970

2. Ma J, Zhang Y, Cichocki A, Matsuno F. A novel EOG/EEG hybrid human–machine interface adopting eye movements and ERPs: application to robot control. IEEE Trans Biomed Eng 2014;62(3):876–889.

3. Adali T, Haykin S. Adaptive signal processing: next generation solutions. Wiley-IEEE Press; 2010. [doi: 10.1002/9780470575758]ISBN:9780470195178

4. Fitzgibbon SP, DeLosAngeles D, Lewis TW, Powers DMW, Grummett TS, Whitham EM, Ward LM, Willoughby JO, Pope KJ. Automatic determination of EMG-contaminated components and validation of independent component analysis using EEG during pharmacologic paralysis. Clin Neurophysiol 2016 Mar;127(3):1781–1793. [doi: 10.1016/j.clinph.2015.12.009]

5. Azami H, Rostaghi M, Abásolo D, Escudero J. Refined composite multiscale dispersion entropy and its application to biomedical signals. IEEE Trans Biomed Eng 2017 Dec;64(12):2872–2879. [doi: 10.1109/TBME.2017.2679136]

6. Task Force of the European Society of Cardiology and the North American Society of Pacing and Electrophysiology. Heart rate variability: Standards of measurement, physiological interpretation, and clinical use. Eur Heart J 1996;17(3):354–381.

7. Boucsein W. Electrodermal Activity. 2nd ed. Springer; 2012.

8. Duchowski AT. Eye tracking methodology. Eye Track Methodol. Springer; 2017. PMID:25246403ISBN:978-3-319-57881-1

9. Kim TT, Lee G. Hospitality employee knowledge-sharing behaviors in the relationship between goal orientations and service innovative behavior. Int J Hosp Manag 2013 Sep;34:324–337. [doi: 10.1016/j.ijhm.2013.04.009]

10. Carver CS, White TL. Behavioral inhibition, behavioral activation, and affective responses to impending reward and punishment: The BIS/BAS Scales. J Pers Soc Psychol 1994;67(2):319–333. [doi: 10.1037/0022-3514.67.2.319]

11. Hsia L-H, Huang I, Hwang G-J. Effects of different online peer-feedback approaches on students’ performance skills, motivation and self-efficacy in a dance course. Comput Educ 2016 May;96:55–71. [doi: 10.1016/j.compedu.2016.02.004]

12. Gosling SD, Rentfrow PJ, Swann Jr. WB. A very brief measure of the Big-Five personality domains. J Res Pers 2003;37:504–528. [doi: 10.1016/S0092-6566(03)00046-1]
